# Public Investment in Animal Protection Work: Data from Manitoba, Canada

**DOI:** 10.3390/ani10030516

**Published:** 2020-03-19

**Authors:** Kendra Coulter, Brittany Campbell

**Affiliations:** 1 Department of Labour Studies, Brock University, St. Catharines, ON L2S 3A1, Canada; 2 Department of Sociology and Anthropology, Carleton University, Ottawa ON K1S 5B6, Canada; brittanycampbell@cmail.carleton.ca

**Keywords:** animal welfare, animal cruelty, animal abuse, animal protection, animals in public policy, animal ethics, humane law enforcement, humane jobs, animals and society, animals and law

## Abstract

**Simple Summary:**

This paper explains how animal protection work is organized and undertaken in Manitoba, Canada. In most Canadian provinces (and countries of the Commonwealth), responsibility for investigations into crimes against animals has been assigned to charities reliant on donations and fundraising. Manitoba is one of the only provinces in Canada to use public money to fund animal cruelty investigations. However, there is no scholarly research on Manitoba’s model. This paper offers the first examination of Manitoba’s publicly funded animal protection model. It explains the organizational structure and investigations process, then identifies strengths and areas for improvement.

**Abstract:**

There is a dearth of research on animal cruelty investigations policy and work, despite its importance for protecting animals from illegal forms of cruelty. This study provides baseline data about the approach used in Manitoba, one of the only Canadian provinces where animal protection is publicly funded. By integrating statistical and qualitative data collected through interviews with key informants, this paper elucidates how animal cruelty investigations are organized and undertaken in the province. Although animal protection in Manitoba is publicly funded, the workforce responsible for undertaking investigations is a cross-section of public and private actors with different occupational classifications and working conditions.

## 1. Introduction

National and regional animal welfare legislation and policy outline minimum standards of care, define which practices are legally prohibited, and establish the framework for investigating suspected animal cruelty. In many European countries and some in the global south (such as Colombia), animal protection is the responsibility of public agencies and either dedicated or general policing services. However, in the Commonwealth countries of the global north such as Canada, the United Kingdom, Australia, and New Zealand, responsibility for front-line enforcement of animal welfare has predominantly fallen to SPCAs (Societies for the Prevention of Cruelty to Animals) and humane societies, nonprofits which depend on donations and fundraising. The off-loading of animal cruelty investigations to charities is atypical; other kinds of law enforcement in these countries are undertaken by police and other public agencies [1,2,3,4,5]. 

SPCAs and humane societies are motivated by a commitment to protecting animals but have smaller workforces and less resources, funding, and enforcement tools than public policing agencies. This makes enforcement work more challenging, increases the physical and psychological risks for officers, and constrains their abilities to reach and most effectively protect animals [6,7,8,9]. 

Effective animal cruelty investigations are significant, first and foremost, because animals are sentient beings who deserve protection [10,11,12,13]. Moreover, a growing body of research has identified a clear link between violence against animals and the simultaneous and/or subsequent abuse of people (especially women and children), and the use of animals to control and harm human victims [14,15,16,17,18,19]. The precise rates vary depending on the jurisdiction, but there is a clear and consistent link between the abuse of animals and people). More law enforcement agencies are also recognizing animal abuse as a “gateway” to other kinds of serious crimes; therefore, there are public safety implications, simultaneously [20,21]. 

Manitoba is one of the only Canadian provinces (and jurisdictions within the Commonwealth) which has a publicly funded animal protection system, rather than charity-based and donation-dependent enforcement [22]. Yet, there is a dearth of research on its policy and approach. Here, we begin to fill this gap. We explain how animal cruelty is investigated in Manitoba and pay particular attention to the organizational structure and resulting investigations process. Animal protection work in Manitoba is coordinated and overseen by the Chief Veterinary Office (CVO), which is housed within the Ministry of Agriculture. However, Manitoba’s approach is not actually a fully public enforcement model but rather a publicly funded and public–private hybrid delivery approach. We assess its strengths and identify areas for improvement. 

## 2. Materials and Methods 

This case study is part of a larger mixed- and multi-method research project examining animal protection policy and work nationally and internationally. We conduct case studies and the data are analyzed in order to assess the efficacy of policy and practice for a) animals (and different groups of animals), b) the front-line workforce (diverse officers as well as dispatchers, veterinary staff, and others providing animal transportation, care, and support), and c) the public (including vulnerable groups of people, human victims of violence, and those being investigated). The organizational structures/investigation agencies we have been studying include SPCAs/humane societies, municipal/local/county animal care and control services, general police forces, and dedicated animal protection units (housed either within larger police forces, or as stand-alone forces). Manitoba is an example of the final category.

Two primary types of data were collected for the Manitoba case study. First, documentary sources, namely provincial legislation and budgets, and other texts produced by the Chief Veterinary Office and Ministry of Agriculture were compiled and examined to provide foundational data. These data were supplemented with semi-structured qualitative interviews. Ten interviews were conducted with key informants who work directly for or are contracted by the Chief Veterinary Office (see Appendix A for the interview guide). Interviews were between 45 and 90 minutes in length.

In keeping with qualitative research conventions, interviews were transcribed and analyzed. They were first read descriptively then thematically, in order to categorize data and identify the most salient patterns [23]. Given the lack of research on animal protection in Manitoba, interviews were essential for building deeper understanding and triangulating the textual data sources by providing greater clarity and detail, as well as the perspectives of those directly involved in enforcement. 

In combination, these two data sets allow us to explain and provide baseline data about Manitoba’s model and are interwoven throughout this paper. 

## 3. Results

Manitoba is a prairie province in central Canada with a population of 1.4 million people. The provincial budget is approximately $17B (CDN). Manitoba, like other Canadian provinces, has provincial animal welfare legislation. Manitoba’s Animal Care Act was created in 1995, and it establishes standards of care, prohibited actions, exclusions (such as for generally accepted agricultural practices), and protocols for investigations. Canada’s federal Criminal Code also contains certain animal cruelty provisions, and these can be used in any province, including Manitoba, where warranted [24]. 

The five main violations outlined in the Animal Care Act are:

1. [Act] 2 (1) (a): Failure to ensure an adequate source of food and water for an animal,
2. [Act] 2 (1) (b): Failure to provide adequate medical attention for an animal when it is wounded or ill,
3. [Act] 2 (1) (c): Failure to provide an animal with reasonable protection from injurious heat or cold,
4. [Act] (2) (1) (d) (ii): Confinement of an animal to an enclosure or area with unsanitary conditions, so as to significantly impair the animal’s health or well-being, and
5. [Act] (3) (1): Inflict upon an animal acute suffering, serious injury or harm, or extreme anxiety or distress that significantly impairs its health or well-being.

### 3.1. Manitoba’s Chief Veterinary Office 

The original Animal Care Act created in 1996, allowed for appointment of Animal Protection Officers (APOs) hired by the province through the Ministry of Agriculture. However, in 2005, the social democratic New Democratic (NDP) government created the Chief Veterinary Office (CVO) to lead animal protection efforts in the province. Then Minister of Agriculture, Rosann Wowchuk, named Dr. Wayne Lees the first Chief Veterinary Officer for Manitoba to oversee all programs run by the CVO [25]. The CVO was created with four specific goals in mind:

1. Protect the health of the public from diseases of animals that can pass directly or indirectly to people.
2. Protect the safety of food to guard against contamination with pathogens, toxins or hazardous materials.
3. Protect the health and welfare of animals for economic or intrinsic benefit.
4. Protect trade in agriculture through health certification or food safety assurance programs [26] (p. 3).

In the formation of the CVO, Manitoba Agriculture recognized core principles of One Health, namely the interconnections between human and veterinary medicine, as well as “the strong inter-relationships among protecting the health of animals, protecting the safety of food, and protecting the health of people” [26] (p. 6). It would be helpful to know more about why specifically animal cruelty investigations were brought under the public funding envelope, including whether it was internally or externally motivated (or some combination), and whether there was any opposition. Unfortunately, we were not able to locate pertinent textual sources or gain insight from the key informants about these historical particulars. 

The CVO has the authority to appoint APOs who are empowered to enforce provincial animal welfare legislation including by conducting investigations, compelling owners to act or to change their behaviour, seizing animals, and/or laying provincial charges. Only police can lay charges under Canada’s Criminal Code, but APOs can lay provincial charges. APOs normally have either an animal-related or law enforcement background and are given eight hours of training.

#### 3.1.1. Organizational Structure: Animal Care Line

Like most jurisdictions, Manitoba relies primarily on complaints from members of the public about suspected animal abuse or neglect. A central Animal Care Line (which receives complaints by phone or by emailed form) has been created to streamline the reporting process. Some of the dispatchers who receive complaints are themselves APOs.

Once a report of animal cruelty has been received, dispatchers at the Animal Care Line assign an APO to investigate. Generally, these assignments are based on the geographic location of APOs in proximity to the location of the complaint. However, these investigations are also often assigned to APOs based on their knowledge and animal preferences. For example, some APOs have previous experience with farmed animals and are more knowledgeable about and comfortable with investigating on farms than others. Workers answering the Animal Care Line are familiar with the available officers and seek out specific investigators accordingly.

#### 3.1.2. Organizational Structure: Animal Protection Officers

**Figure 1:**
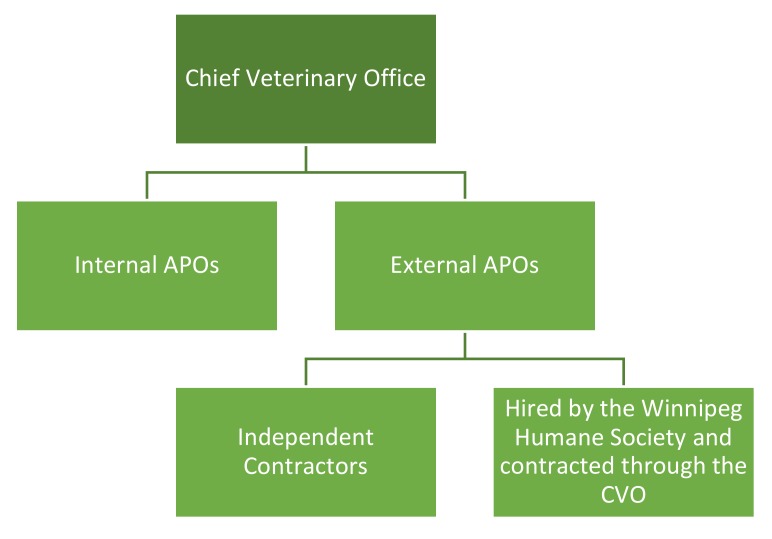
Types of Animal Protection Officers (APOs) in Manitoba.

There are different groupings of APOs. Internal APOs are direct, public, government employees. External APOs are contracted to undertake investigations. External APOs fit into two further categories: independent contractors and those who work for the Winnipeg Humane Society but are appointed by the CVO and focus on cruelty investigations (Figure 1). Under Canadian laws, independent contractors, even though they are people, are legally considered to be individual *businesses* that are under contract to another organization or business. As a result, APOs who are independent contractors are not legally classified as employees and are exempt from most labour laws and employment standards. 

In total, there are about 105 APOs in Manitoba, and the split is approximately 60% external and 40% internal. Not all APOs are responsible for front-line investigations on a full-time basis or as a primary responsibility. Internal APOs, the direct employees of the CVO, include veterinarians, program supervisors, and other office staff, including dispatchers. They often have other primary work duties and are called upon to directly undertake or assist with animal cruelty investigations less frequently. 

The CVO also appoints people outside of the ministry to serve as APOs and conduct animal cruelty investigation work. These external APOs work on a case-by-case basis; many have full-time careers and only work part-time on investigations. In keeping with convention for independent contractors, these APOs log their activity and are paid hourly for their services, travel mileage, phone calls, etc. 

The second group of external APOs appointed by the CVO work for the Winnipeg Humane Society (WHS) and are responsible for front-line investigations within the city. In 2011, the WHS created the Department of Investigations and Emergency Response and is the primary animal welfare organization in Winnipeg. This is the only animal charity in the province that has a provincially appointed cruelty investigation team. Winnipeg is the provincial capital and home to 832,186 people, close to 2/3 of Manitoba’s total population [27]. There are four full-time and one part-time APOs for the city of Winnipeg.

The WHS also employs emergency responders (ERs) to help handle animal emergencies and reports of cruelty in Winnipeg. ERs are not classified as officers under the Animal Care Act and have limited enforcement power, but they can travel in the field with APOs and assist with investigations in specific ways, particularly by interacting with members of the public while on scene and watching for risks or threats to the officers. ERs can be recommended by management to be appointed as APOs through the Ministry of Agriculture. These workers may then be hired by the WHS or by the CVO as APOs. 

APOs in Manitoba are compared below in Table 1. 

There are clear inequities in compensation, labour rights and protections, equipment, and transportation among these groups of APOs. We consider the implications further below.

### 3.2. The Investigations Process

**Figure 2:**
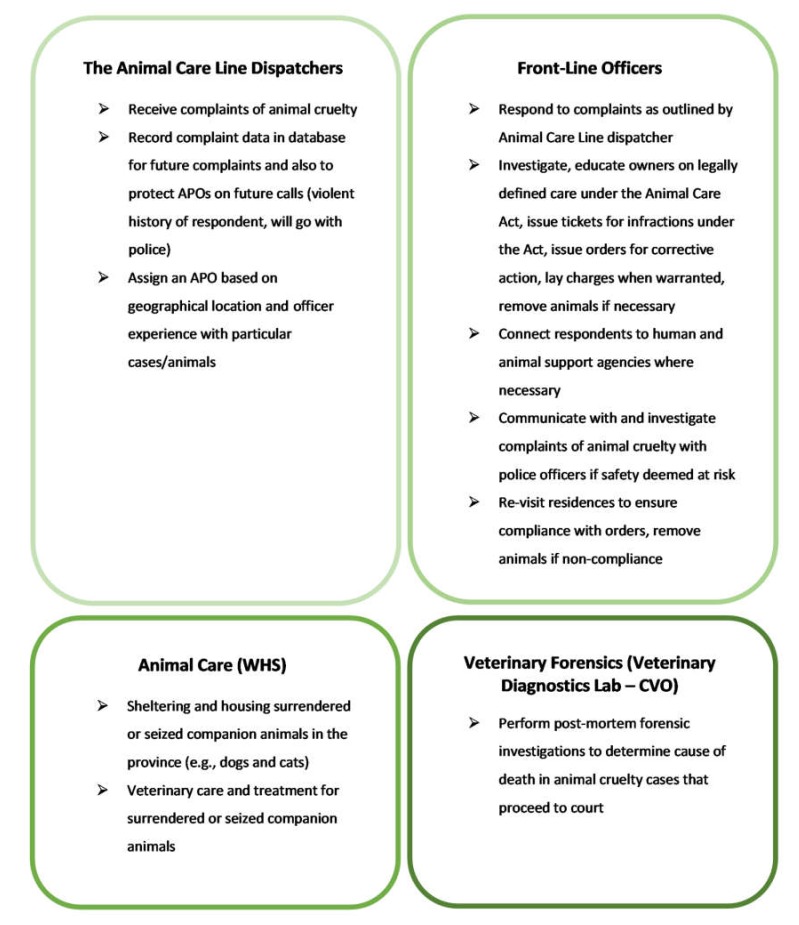
Basic Process of Manitoba Cruelty Investigations.

Succinctly, the animal cruelty investigations process (see Figure 2) in Manitoba involves the following components: 

As noted, Animal Care Line dispatchers are familiar with the pool of APOs and can seek out specific investigators accordingly. APOs can then decide to accept or decline an investigation request. This is highly unusual in animal cruelty investigation and other kinds of law enforcement work. Yet, because the majority of APOs in the province are external staff/contractors with other jobs and responsibilities, they may not always be available to investigate all reports of cruelty or to do so promptly. 

During an investigation, APOs seek to determine whether an animal owner or caretaker is in compliance with the Animal Care Act and recommend the appropriate action. The CVO responds to reports of cruelty for both farmed and companion animals and uses the term *inspection* for all complaints that are investigated, whether it be at a business or a residence. This is somewhat different from many other jurisdictions where the term inspection is more commonly applied to the proactive examination of businesses, while investigations are individual, complaints-based cases. Potential results of an inspection in Manitoba are outlined in Table 2. 

After an investigation, APOs follow up with dispatchers to provide the result(s) of the investigation. A database is maintained which benefits investigators if there are future complaints at the same location. The WHS, which receives reports of abuse through the Animal Care Line as well as its own animal welfare line, also maintains its own database.

The CVO generates annual statistics on investigations (see Table 3). 

Despite the varying percentage of total cases examined per year, this data shows that Act 2 (1) (a) of the Animal Care Act is violated the most (standards of care), and that canines are consistently the most inspected species.

The WHS also keeps statistical records with some overlapping and some distinct data (Table 4). 

The number of complaints has more than tripled since 2009. We note that the increase of animal cruelty cases annually (for both the CVO and the WHS) may or may not reflect increased animal mistreatment in the province. It may be because the public has become more aware of animal welfare and can easily report suspected violations through the Animal Care Line. 

The CVO also records investigation outcomes. Table 5 outlines the actions that can be taken by APOs after finding non-compliance with the Animal Care Act. 

Table 6 and Table 7 provide details about the frequency of inspection outcomes. The number of tickets issued was not identified in the data set after 2016. Percentages may exceed 100% as cases may involve more than one outcome simultaneously. 

It would be helpful for assessing investigations and results over time if there were consistency in reporting between the CVO and WHS and if there were one central database that includes the number of corrective actions assigned, animals seized, and charges laid by all APOs. Nevertheless, these figures are of use for building an understanding of the types of infractions and the resulting steps taken.

Each year, the greatest number of calls the WHS receive are emergency pick-ups of injured animals, and this clearly takes up a great deal of APO time. In Canada, this kind of work is normally undertaken by municipal/local animal control services and seen as distinct from animal cruelty investigations in most jurisdictions [28]. Municipalities often outsource these kinds of animal care and control tasks to private organizations, including nonprofits [9].

In terms of animal cruelty investigations and the enforcement of the provincial Animal Care Act, both data sets reveal that APOs investigate a large number of suspected violations of Section 2 (1)(a) of the provincial act: failure to ensure adequate source of food or water. APOs in Winnipeg also investigate many suspected violations of Section 2 (1) (c): failure to provide an animal with reasonable protection from injurious heat or cold. The latter is significant because Manitoba has harsh winters. These standards of care violations may result from caregiver indifference or they may stem from a lack of knowledge or a lack of resources. What little data exist reveal that standards of care violations are common across jurisdictions [9,29]. 

We have not undertaken a detailed comparative analysis of the statistical data on types of enforcement responses and outcomes, and how they are related to different investigation models in this paper. Given our focus and objectives, this would be a different undertaking, albeit one that is linked and worthy. We note that the United States Federal Bureau of Investigation is now tracking felony animal abuse crimes. Unfortunately, Canada’s national statistics agency does not currently collect data about animal cruelty. The centralized gathering of animal investigations crimes data by Statistics Canada would be highly valuable. 

### 3.3. Key Qualitative Findings

The qualitative data from Manitoba reinforce the findings from the small body of research on animal cruelty investigations which have consistently found that officers engage daily in multiple kinds of labour and require a cross-section of skills [6,8]. Coulter has argued that animal protection officers are part law enforcement, part social worker, and part nurse [9,30]. Officers also educate many individual members of the public about animal care and available resources and supports during the investigations process [31]. 

Succinctly, *investigations* in a full sense are needed to determine if something illegal is occurring, and, if so, how, why, and what the most appropriate response(s) would be. As noted above, officers have multiple tools available to them ranging from recommending a change in behavior (which is most common) to seizure of animals and more serious criminal justice mechanisms such as the laying of charges under the provincial Animal Care Act. APOs can also involve the police when other crimes are discovered or if an officer believes charges under Canada’s Criminal Code are warranted. While conducting investigations, officers interact with people in a range of socioeconomic situations including poverty and those confronting housing, health, and/or mental health issues. Hoarding of animals is especially complex and results from a mental health disorder [32,33]. As noted above, animal cruelty is increasingly recognized as connected to the abuse of people, particularly women and children, and as a gateway to other crimes of crimes. These dimensions augment the challenge and point to the need for cross-reporting, additional data collection, and greater collaboration among animal protection officers, social service providers, health care workers, and other sorts of supportive organizations. Animal protection work is an opportunity to improve the wellbeing of people and animals alike, when resources are available. 

Officers must be prepared to discover many kinds of situations and to react accordingly. This work, wherever it is undertaken, is challenging and risky—physically, psychologically, and emotionally. The APOs in Manitoba highlighted the need for more effective mental health supports administered by professionals who are familiar with and knowledgeable about the particular challenges first responders must confront daily. We note that this is a frequent comment shared by officers across our field sites [7,9].

## 4. Discussion

Overall, Manitoba’s approach is different from the dominant model used in most Canadian provinces (and countries of the Commonwealth), wherein responsibility is off-loaded to nonprofits that fund investigations through their own donation-dependent budgets. The Manitoba government provides public funding for animal welfare law enforcement within the province which is laudable. There are compelling ethical, human safety, health, feminist, and workers’ rights reasons for governments to be investing in animal cruelty investigations. Manitoba has been a path-maker in this respect and had the foresight to see the multifaceted importance of developing public policy that reflects the multispecies nature of our families, community, and society and assigning public funds to reflect these realities. 

However, this is not a fully public investigations model because of the heavy reliance on external contractors. Manitoba’s model involves public funding but a hybrid of public and private actors undertaking front-line investigations. In most cases, APOs are either independent contractors or employees of a non-profit, contracted to undertake investigations. The centralized Animal Care Line helps streamline the reporting process for members of the public and is an important component of the province’s approach. However, in the field, the differences become particularly salient. 

The patchwork of service around the province results in inconsistent coverage for different communities, and noteworthy occupational inconsistencies across the three types of APOs. Having three different types of APOs creates and reproduces significant inequities in all the major categories of working conditions: compensation, employment rights, labour protections, protective, equipment, and transportation. The working conditions, caseloads, travel times, and resources available to APOs affect officer morale, safety, and wellbeing. Such conditions also affect how quickly APOs can reach animals, how long they have for investigations, and how thorough they can be. These are occupations where human and animal wellbeing are directly linked: the size and effectiveness of the workforce affects the efficacy of animal protection [9,30]. It is also noteworthy that as independent contractors, external APOs can choose to accept or not accept a case based on their availability which adds to the length of time an animal might remain in distress. This is not ideal.

## 5. Conclusions

In the interest of developing a more robust and consistent approach to animal protection that promotes equity for APOs and consistency in service for communities around the province, we recommend that Manitoba move to a fully public model of investigations which involves a dedicated unit of APOs who are all government employees. We note that the province of Ontario, Canada’s largest, has just recently passed legislation to create such a fully public dedicated animal protection team. 

One consolidated force of APOs would eliminate the occupational inequities, ensure more consistent and time-sensitive responses around the province, and improve the efficacy of investigations. It would also facilitate centralized reporting and management of data which can help inform future policy making, foster greater and more nuanced assessment of the results of enforcement (including harm prevention), and improve officer safety.

In that spirit, we recommend that the training provided to APOs be increased from the current eight hours and that the province consult with APOs directly, the MGEU, other enforcement agencies, and health care specialists in order to develop a strong mental health support program for APOs. We also recommend additional engagement with human-focused law enforcement agencies in Manitoba in order to ensure local and federal police working within the province understand animal protection processes and resources, and are trained in understanding the human–animal violence link. Ideally, such training and collaboration would also extend to and include child protection workers and those in the domestic violence sector.

**Figure 3:**
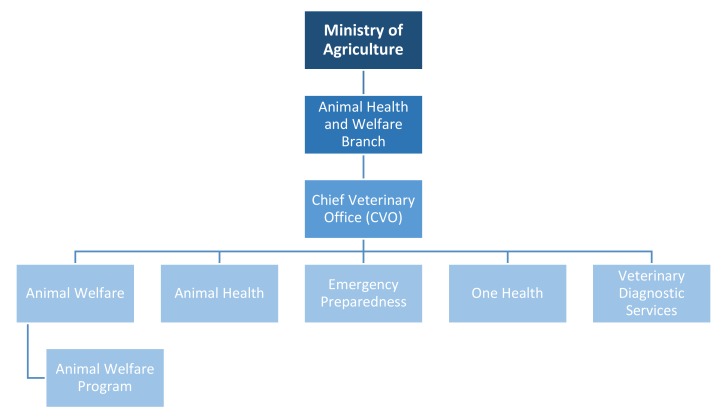
Ministry Structure and Initiatives.

Notably, animal protection encompasses more than cruelty investigations in the province (see Figure 3). 

This multi-dimensional approach recognizes the distinct and interconnected realities of human and animal health and wellbeing, the need for responsive and proactive policies and programs, and the multispecies nature of our societies. We encourage ongoing research, analysis, and attentive policy making as these complementary areas are further developed and refined. 

Globally, there is a need for much more research on investigations and animal protection work, both quantitative and qualitative, in order to identify challenges, areas for improvement, and best practices, with the goals of better protecting animals, officers, vulnerable people and communities, and the public overall. 

## Figures and Tables

**Table 1:** Comparing Types of APOs in Manitoba.

Internal APOs (Employees of CVO—Chief Veterinary Office)	External APOs (Independent Contractors)	External APOs (Working for the WHS—Winnipeg Humane Society)
CVO staff appointed as APOs	Hired as independent contractors	Hired directly by WHS as ERs then get promoted to APOs and contracted by the CVO to conduct investigations in Winnipeg
Work full-time within the CVO in various positions and complete investigations as part of their jobs if/when necessary	Work part-time (a secondary career for most APOs)	Unionized as part of the Canadian Union of Public Employees
Salaried	Paid hourly for services (must log work hours and tasks)	Paid hourly
Unionized and part of the Manitoba Government and General Employees’ Union (MGEU)	No benefits provided by CVO	Have access to therapist three times a year
Receive benefits through EFAP (Employee and Family Assistance Program)	Use personal vehicles for investigations	Travel in WHS investigation vehicles
Government vehicles for inspections	No required uniform; carry an APO identification card	Wear WHS uniform

**Table 2:** Results of Animal Cruelty Inspections as Outlined by the CVO.

Dismissal	A Concern is Dismissed if the Inspection Produces no Evidence of Abuse or Animals in Distress.
Corrective Action	For minor infractions, the APO outlines improvements the owner must make. A follow-up inspection is performed to ensure the owner has complied.
Seizure of Animals	If there are reasonable grounds to believe animals are in distress, the APO may supply any care deemed necessary to relieve the distress. Under Section 9 (1) of the Act, the APO may also seize the animals, either immediately or at a later date. Seizure of animals is for the purpose of protecting the animals and relieving distress and is not a form of punishment of the owner.
Charges Under the Animal Care Act	If infractions to the Animal Care Act are discovered, the matter is investigated, and charges may be filed. Charges may include: Common Offence Notice (CON)/fines Court prosecution

Data supplied by Manitoba Agriculture.

**Table 3:** Chief Veterinary Office Animal Cruelty Statistics between 2013 and 2019.

Year	Total Cases Filed	Largest Animal Welfare Concern	Most Inspected Species
2013	582	N/A	Canine (47%)
2014	696	N/A	Canine (64.9%)
2015	798	[Act] 2 (1) (a) Failure to ensure adequate source of food and water for an animal (51.75%)	Canine (68.3%)
2016	952	[Act] 2 (1) (a) Failure to ensure adequate source of food and water for an animal (43.8%)	Canine (66.81%)
2017	1026	[Act] 2 (1) (a) Failure to ensure adequate source of food and water for an animal (52%)	Canine (64%)
2018	1054	[Act] 2 (1) (a) Failure to ensure adequate source of food and water for an animal (52%)	Canine (66%)
2019	809	[Act] 2 (1) (a) Failure to ensure adequate source of food and water for an animal (50%)	Canine (67%)

Data supplied by Manitoba Agriculture.

**Table 4:** Winnipeg Humane Society Animal Cruelty Statistics between 2014 and 2018.

Year	Total Cases Filed	Case Breakdown
2014	739	108 injured or ill wildlife emergency pick-ups 190 animals locked in vehicle complaints 34 confinement complaints regarding inadequate ventilation/lighting 407 calls regarding animals unduly exposed to heat/cold
2015	1832	625 emergency pick-ups 226 animals locked in vehicles complaints 375 calls of complaint for not providing enough food or water 435 calls regarding animals unduly exposed to cold or heat 171 animals abandoned or living in conditions causing extreme anxiety/distress
2016	2264	952 emergency pick-ups 185 animals locked in vehicles complaints 474 calls of complaint for not providing food or water 484 calls regarding animals unduly exposed to cold or heat 169 calls regarding abandoned animals
2017	2597	324 animals locked in vehicles complaints 543 calls of complaint for not providing food or water 970 emergency pick-ups 228 calls regarding abandoned animals 532 calls regarding animals unduly exposed to cold or heat
2018	2918	1737 welfare cases attended 669 emergency calls 512 non-emergency calls

Data supplied by the Winnipeg Humane Society.

**Table 5:** Actions Taken by APOs if non-compliance with Animal Care Act.

Recommendation	APO Makes Recommendations to Owner to Achieve Compliance with the Animal Care Act.
Compliance Following Recommendations	APO finds owner to be providing care in compliance with the Animal Care Act after recommendations were made by an APO during a previous inspection.
Surrender	Owner transfers all rights of ownership of animal to APO.
Director’s Order	APO finds owner repeatedly non-compliant with the Animal Care Act. Order issued by director of Animal Care Act to enforce compliance with animal care guidelines of the Animal Care Act and/or improvements need to be made to animal care immediately to prevent animal suffering.
Issued Notice of Seizure/Custody	APO issues notice of seizure/custody to owner when: (1) animal is deemed to be in distress and requires medical intervention, (2) an owner is non-compliant with an order, or (3) an animal is abandoned and taken into custody to receive care.
Issued Notice of Distress	APO issues notice of distress to owner when animal is suffering to a degree where it is inhumane to allow them to continue to live. Animal is seized by APO, and humanely euthanized.

Data supplied by Manitoba Agriculture.

**Table 6:** Chief Veterinary Office Inspection Outcomes where Non-Compliance is Identified, 2013–2016 (Percentages).

Year	Corrective Actions Taken	Complaint Dismissed	Surrender	Seized	Tickets Issued/Prosecuted	Order
2013	48	31	9	5	1	4
2014	86.6	31.2	16.5	6.8	2.6	6.6
2015	33.08	28.7	13.66	4.51	3.01	2.26
2016	32.77	52.84	16.91	6.83	1.79	3.36

Data supplied by Manitoba Agriculture.

**Table 7:** Chief Veterinary Office Inspection Outcomes where Non-Compliance is Identified, 2017–2019 (Percentages).

Year	Recommendation	Compliance Following Recommendations	Surrender	Director’s Order	Issued Notice of Seizure/Custody	Issued Notice of Distress
2017	84	64	37	11	14	0.8
2018	59	51	22	10	14	0.4
2019	59	51	22	10	14	0.4

Data supplied by Manitoba Agriculture.

## Appendix A

**Interview Guide**

1. In your own words, can you please describe the Chief Veterinary Office, its mandate, goals, and services they provide?
2. From your perspective, what are some of the benefits of the CVO?
3. What are some of the limitations of the CVO?
4. Can you tell me about your current occupation or role within the CVO?
  a. What is a typical day at work for you?
5. How long have you been employed with the CVO?
  a. Were you employed in animal cruelty investigation or a similar field prior to working with the CVO?
  b. If yes, how do your experiences at your old job differ from working at the CVO?
6. What do you like most about your job?
7. What do you dislike most about your job?
8. Do any specific instances stand out for you and why?
9. If you are comfortable, can you please comment on the compensation and benefits you receive as an animal cruelty investigator employed by the CVO?
10. What would you like to see addressed/changed within the CVO?
  a. What would help improve your ability to do your job?
11. Are there any projected changes for the CVO?
12. Is there anything else you would like to share regarding your work within the CVO, or the CVO more broadly, that was not addressed in this interview?
13. If I need to clarify something you said during this interview, may I have your permission to contact you for a follow-up phone call?
